# *Notes from the Field:* Comparison of COVID-19 Mortality Rates Among Adults Aged ≥65 Years Who Were Unvaccinated and Those Who Received a Bivalent Booster Dose Within the Preceding 6 Months — 20 U.S. Jurisdictions, September 18, 2022–April 1, 2023

**DOI:** 10.15585/mmwr.mm7224a6

**Published:** 2023-06-16

**Authors:** Amelia G. Johnson, Lauren Linde, Amanda B. Payne, Akilah R. Ali, Vanessa Aden, Brandy Armstrong, Brett Armstrong, Steven Auche, Nagla S. Bayoumi, Sarah Bennett, Rachelle Boulton, Carolyn Chang, Abigail Collingwood, Kevin Cueto, Sherri L. Davidson, Yi Du, Aaron Fleischauer, Victoria Force, Darren Frank, Ross Hamilton, Kaitlin Harame, Pauline Harrington, Liam Hicks, Jeffrey D. Hodis, Mikhail Hoskins, Amanda Jones, FNU Kanishka, Ramandeep Kaur, Samantha Kirkendall, Saadiah I. Khan, Anna Klioueva, Ruth Link-Gelles, Shelby Lyons, Joshua Mansfield, Amanda Markelz, John Masarik, Erica Mendoza, Keeley Morris, Enaholo Omoike, Sai Paritala, Komal Patel, Melissa Pike, Xandy Peterson Pompa, Kevin Praetorius, Nadine Rammouni, Hilda Razzaghi, Alexa Riggs, Minchan Shi, Nekabari Sigalo, Emma Stanislawski, Buddhi P. Tilakaratne, Kathryn A. Turner, Caleb Wiedeman, Benjamin J. Silk, Heather M. Scobie

**Affiliations:** ^1^COVID-19 and Other Respiratory Viruses Division, National Center for Immunization and Respiratory Diseases, CDC; ^2^Georgia Department of Public Health; ^3^West Virginia Department of Health and Human Resources; ^4^Kentucky Department for Public Health; ^5^Council of State and Territorial Epidemiologists, Atlanta, Georgia; ^6^New Jersey Department of Health; ^7^Indiana Department of Health; ^8^Utah Department of Health and Human Services; ^9^New York City Department of Health and Mental Hygiene, New York, New York; ^10^Nebraska Department of Health and Human Services; ^11^Alabama Department of Public Health; ^12^North Carolina Department of Health and Human Services; ^13^CDC COVID-19 Emergency Response Team; ^14^Booz Allen Hamilton, Inc., McLean, Virginia; ^15^Colorado Department of Public Health & Environment; ^16^Michigan Department of Health and Human Services; ^17^Arizona Department of Health Services; ^18^Center for Surveillance, Epidemiology and Laboratory Services, CDC; ^19^Idaho Department of Health and Welfare; ^20^Texas Department of State Health Services; ^21^Louisiana Department of Health; ^22^Minnesota Department of Health; ^23^District of Columbia Department of Health, Washington, DC; ^24^New Mexico Department of Health; ^25^CDC Foundation, Atlanta, Georgia; ^26^Tennessee Department of Health; ^27^Immunization Services Division, National Center for Immunization and Respiratory Diseases, CDC.

Updated (bivalent) COVID-19 vaccines were first recommended by CDC on September 1, 2022.* An analysis of case and death rates by vaccination status shortly after authorization of bivalent COVID-19 vaccines showed that receipt of a bivalent booster dose provided additional protection against SARS-CoV-2 infection and associated death (*1*). In this follow-up report on the durability of bivalent booster protection against death among adults aged ≥65 years, mortality rate ratios (RRs) were estimated among unvaccinated persons and those who received a bivalent booster dose by time since vaccination during three periods of Omicron lineage predominance (BA.5 [September 18–November 5, 2022], BQ.1/BQ.1.1 [November 6, 2022–January 21, 2023], and XBB.1.5 [January 22–April 1, 2023]).^†^

During September 18, 2022–April 1, 2023, weekly counts of COVID-19–associated deaths^§^ among unvaccinated persons and those who received a bivalent booster dose^¶^ were reported from 20 U.S. jurisdictions** that routinely link case surveillance data to immunization registries and vital registration databases (*1*). Vaccinated persons who did not receive a bivalent COVID-19 booster dose were excluded. Rate denominators were calculated from vaccine administration data and 2019 U.S. intercensal population estimates,^††^ with numbers of unvaccinated persons estimated by subtracting numbers of vaccinated persons from the 2019 intercensal population estimates, as previously described^§§^ (*1*). Average weekly mortality rates were estimated based on date of specimen collection^¶¶^ during each variant period by vaccination status and time since bivalent booster dose receipt. RRs were calculated by dividing rates among unvaccinated persons by rates among bivalent booster dose recipients; after detrending the underlying linear changes in weekly rates, 95% CIs were estimated from the remaining variation in rates observed*** (*1*). SAS (version 9.4; SAS Institute) and R (version 4.1.2; R Foundation) software were used to conduct all analyses. This activity was reviewed by CDC and was conducted consistent with applicable federal law and CDC policy.^†††^

Among adults aged ≥65 years who were unvaccinated or had received a bivalent COVID-19 vaccine booster dose, 8,161 COVID-19–associated deaths in the 20 U.S. jurisdictions were reported during September 18, 2022–April 1, 2023. Overall, 58% of deaths occurred among adults aged ≥80 years; this distribution was consistent among vaccinated and unvaccinated persons and across the three variant periods. Mortality rates among adults aged ≥65 years peaked in December 2022 during the BQ.1/BQ.1.1 predominance period. Higher mortality rates were observed among unvaccinated persons during all three periods of Omicron lineage predominance (Table).

**TABLE:** Average weekly mortality rates* and rate ratios for unvaccinated adults aged ≥65 years compared with those vaccinated with a bivalent COVID-19 vaccine booster dose,^†^ by time since vaccination and variant period^§^ — 20 U.S. jurisdictions,^¶^ September 18, 2022–April 1, 2023

Period (predominant Omicron lineage)	Total	Vaccination status				
		Unvaccinated	Vaccinated with bivalent booster dose, by time since vaccination**			
			2 wks–2 mos		3–6 mos	
		No. of deaths (mortality rate)	No. of deaths (mortality rate)	RR (95% CI)^††^	No. of deaths (mortality rate)	RR (95% CI)
**Sep 18–Nov 5, 2022 (BA.5)**	**1,717**	1,623 (13.5)	94 (0.8)	16.3 (13.8–19.1)	NA^§§^	NA^§§^
**Nov 6, 2022–Jan 21, 2023 (BQ.1/BQ.1.1)**	**4,537**	3,532 (18.8)	794 (1.6)	11.4 (9.4–13.9)	211 (1.8)	11.0 (8.4–14.4)
**Jan 22–Apr 1, 2023 (XBB.1.5)**	**1,907**	1,247 (7.3)	114 (0.9)	8.4 (6.1–11.7)	546 (1.0)	7.3 (6.1–8.7)

**Abbreviations:** NA = not applicable; RR = rate ratio.

* Deaths per 100,000 persons aged ≥65 years. A COVID-19–associated death occurred in a person with a documented COVID-19 diagnosis who died and whose report local health authorities reviewed to make that determination (e.g., using vital records, public health investigation, or other data sources). Per national guidance, this group should include persons whose death certificate lists COVID-19 or SARS-CoV-2 as an underlying cause or a significant condition contributing to death (https://preparedness.cste.org/wp-content/uploads/2022/12/CSTE-Revised-Classification-of-COVID-19-associated-Deaths.Final_.11.22.22.pdf). In some jurisdictions, deaths that were not laboratory-confirmed were included.

^†^ Unvaccinated persons did not receive any COVID-19 vaccine doses. Persons vaccinated with a bivalent booster dose received a primary series and an authorized bivalent COVID-19 vaccine dose on or after September 1, 2022, and ≥14 days before the positive specimen collection date; bivalent vaccines reported as first or second doses are classified as bivalent booster doses. Reinfections occurring ≥90 days after a previous infection were included.

^§^ National weighted estimates of weekly proportions of circulating SARS-CoV-2 variants are reported by CDC (https://covid.cdc.gov/covid-data-tracker/#variant-proportions). Analysis periods were categorized based on Omicron lineage predominance (>50% of sequenced lineages) for BA.5 (September 18–November 5, 2022) and XBB.1.5 (January 22–April 1, 2023). The BQ.1/BQ.1.1 period (November 6, 2022–January 21, 2023) also included other lineages with similar mutations and was defined based on when BA.5 reached <50% related to the rise of these other lineages.

^¶^ The 20 jurisdictions included in this analysis represent 41% of the overall U.S. population: Alabama, Arizona, Colorado, District of Columbia, Georgia, Idaho, Indiana, Kentucky, Louisiana, Michigan, Minnesota, Nebraska, New Jersey, New Mexico, New York City, North Carolina, Tennessee, Texas, Utah, and West Virginia.

** Time since receipt of last bivalent booster dose categories was restricted to outcomes occurring during eligible weeks based on the timing of the first bivalent booster dose recommendation on September 1, 2022: 2 weeks–2 months (starting September 18, 2022) and 3–6 months (starting December 4, 2022). Unvaccinated persons were compared with persons who received a bivalent booster dose for the same time frame in each category. The median time since vaccination in the 2 weeks–2 months category (14–89 days) was 22 days for the BA.5 period, 58 days for the BQ.1/BQ.1.1 period, and 72 days for the XBB.1.5 period. For the period 3–6 months since vaccination (90–212 days), the median time was 101 days during the BQ.1/BQ.1.1 period and 132 days for the XBB.1.5 period.

^††^ RRs were calculated as the ratio between rates among unvaccinated persons compared with rates among bivalent booster dose recipients by time since vaccination (2 weeks–2 months and 3–6 months). 95% CIs were calculated after detrending underlying linear changes in weekly rates using piecewise linear regression. Each 95% CI represents the remaining variation in observed weekly rates and resulting RRs. The number of observations leading to each 95% CI reflects the number of weeks per period: BA.5 (7 weeks), BQ.1/BQ.1.1 (11 weeks), and XBB.1.5 (10 weeks).

^§§ ^Based on the timing of the authorization of the bivalent vaccine on September 1, 2022.

In time-stratified analyses comparing mortality rates among unvaccinated persons with those among vaccinated persons 2 weeks–2 months after receipt of a bivalent booster dose, mortality RRs significantly declined from 16.3 during the BA.5-predominant period to 8.4 during the XBB.1.5-predominant period, representing a modest reduction in crude vaccine effectiveness from 94% to 88%.^§§§^ Mortality RRs were similar among persons who had received a bivalent booster dose either 2 weeks–2 months earlier or 3–6 months earlier in the BQ.1/BQ.1.1-predominant period (11.4 and 11.0, respectively) and in the XBB.1.5-predominant period (8.4 and 7.3, respectively).^¶¶¶^

The findings in this report are subject to at least six limitations. First, this ecologic study using surveillance data could not adjust for important confounders, such as variations in infection-derived immunity, comorbidities, and testing or prevention behaviors, which might contribute to mortality rate differences by vaccination status. Second, a decrease in mortality during the XBB.1.5 period limited sample sizes and statistical power. Third, only seven of 20 jurisdictions were able to include COVID-19–associated deaths without laboratory-confirmation, which have increased over time.**** Fourth, potential misclassification of bivalent and monovalent booster doses could influence RRs (*2*). Fifth, a small proportion (0.3%) of persons received a second bivalent booster before April 1, 2023, although this was not authorized for some persons until April 19, 2023.^††††^ Finally, these data represent approximately 41% of the U.S. population, and therefore, might not be generalizable.

In this assessment of the durability of protection afforded by a bivalent booster dose against COVID-19–associated death among adults aged ≥65 years, receipt of a bivalent booster dose provided substantial protection, with no significant evidence of waning up to 6 months postvaccination; this finding is similar to other studies assessing vaccine effectiveness against critical illness and death (*1*,*3*,*4*). However, some immune evasion was observed during the Omicron XBB.1.5 period (evidenced by a 6% decrease in vaccine effectiveness compared with that during the BA.5 period), likely related to changes in the spike protein relative to the BA.4/BA.5 spike contained in the bivalent vaccine. These findings are relevant to future decisions on COVID-19 vaccine composition.^§§§§^ With the expiration of the COVID-19 public health emergency declaration on May 11, 2023, routine monitoring of incidence, hospitalization rates, and death rates by vaccination status has been discontinued; however, CDC will continue to monitor vaccine effectiveness through well-controlled studies (e.g., the VISION and IVY networks) (*5*). All persons should stay up to date with recommended COVID-19 vaccines, including ≥1 bivalent dose for persons aged ≥6 months. Additional bivalent vaccine doses are optional for adults aged ≥65 years and immunocompromised persons.

## Footnotes

* https://www.cdc.gov/coronavirus/2019-ncov/vaccines/stay-up-to-date.html

† National weighted estimates of weekly proportions of circulating SARS-CoV-2 variants are reported by CDC (https://covid.cdc.gov/covid-data-tracker/#variant-proportions). Analysis periods were categorized based on Omicron lineage predominance (>50% of sequenced lineages) for BA.5 (September 18–November 5, 2022) and XBB.1.5 (January 22–April 1, 2023). The BQ.1/BQ.1.1 period (November 6, 2022–January 21, 2023) also included other lineages with similar mutations and was defined based on when BA.5 reached <50% related to the rise of these other lineages.

§ A COVID-19–associated death occurred in a person with a documented COVID-19 diagnosis who died and whose report local health authorities reviewed to make that determination (e.g., using vital records, public health investigation, or other data sources). Per national guidance, this group should include persons whose death certificate lists COVID-19 or SARS-CoV-2 as an underlying cause or a significant condition contributing to death (https://preparedness.cste.org/wp-content/uploads/2022/12/CSTE-Revised-Classification-of-COVID-19-associated-Deaths.Final_.11.22.22.pdf). In some jurisdictions, deaths that were not laboratory-confirmed were included.

¶ Unvaccinated persons did not receive any COVID-19 vaccine doses. Persons vaccinated with a bivalent booster dose received a primary series and an authorized bivalent COVID-19 vaccine dose on or after September 1, 2022, and ≥14 days before the positive specimen collection date; bivalent vaccines reported as first or second doses are classified as bivalent booster doses. Reinfections occurring ≥90 days after a previous infection were included.

** The 20 jurisdictions included in this analysis represent 41% of the U.S. population: Alabama, Arizona, Colorado, District of Columbia, Georgia, Idaho, Indiana, Kentucky, Louisiana, Michigan, Minnesota, Nebraska, New Jersey, New Mexico, New York City, North Carolina, Tennessee, Texas, Utah, and West Virginia.

††
https://www.census.gov/programs-surveys/popest/data/tables.2019.html

§§ A continuity correction was applied to denominators by capping vaccination coverage at 95%; it was assumed that ≥5% of each age group would be unvaccinated in each jurisdiction. Adding this correction ensures that there is always a reasonable denominator for the unvaccinated population and prevents incidence and death rates from growing unrealistically large because of potential overestimates of vaccination coverage.

¶¶ For deaths that were not laboratory-confirmed, alternative reference dates were used in lieu of positive specimen collection date (i.e., symptom onset date or date of death or report date when symptom onset date was unavailable).

*** 95% CIs were calculated after detrending underlying linear changes in weekly rates using piecewise linear regression. Each 95% CI represents the remaining variation in observed weekly rates and resulting RRs. The number of observations leading to each 95% CI reflects the number of weeks per period: BA.5 (7 weeks), BQ.1/BQ.1.1 (11 weeks), and XBB.1.5 (10 weeks).

††† 45 C.F.R. part 46.102(l)(2), 21 C.F.R. part 56; 42 U.S.C. Sect. 241(d); 5 U.S.C.0 Sect.552a; 44 U.S.C. Sect. 3501 et seq.

§§§ To interpret RR changes, age group-specific crude vaccine effectiveness was estimated as (1 − [incidence in vaccinated / incidence in unvaccinated]) x 100%.

¶¶¶ The median time since vaccination during the 2 weeks–2 months postvaccination category (14–89 days) was 22 days for the BA.5 period, 58 days for the BQ.1/BQ.1.1 period, and 72 days for the XBB.1.5 period. For the period 3–6 months since vaccination (90–212 days), the median time was 101 days during the BQ.1/BQ.1.1 period and 132 days for the XBB.1.5 period.

****
https://www.cdc.gov/coronavirus/2019-ncov/science/data-review/index.html

††††
https://www.cdc.gov/media/releases/2023/s0419-covid-vaccines.html

§§§§
https://www.who.int/news/item/18-05-2023-statement-on-the-antigen-composition-of-covid-19-vaccines
